# Effect of tea polyphenols as an antioxidant on pork for frying at different temperatures and times

**DOI:** 10.1002/fsn3.3901

**Published:** 2024-01-04

**Authors:** WeiJiang Fan, GuoHui Du, XueNa Zhang, ShuQing Wang, FeiHong Long, Chao Li, NingPeng Jiang, Yinglong Zhang, Qiang Sun

**Affiliations:** ^1^ College of Food Industry Shandong Institute of Commerce and Technology Jinan China; ^2^ Tianjin Food Group Tianjin China; ^3^ Department of Science and Technology of Shandong Province Jinan China

**Keywords:** antioxidants, cooking, frying, tea polyphenols

## Abstract

The aim of this study was to investigate the effect of frying on the antioxidant properties of tea phenols added to pork. The antioxidant capacity of tea polyphenols with different concentrations was tested using different assays including total antioxidant capacity (T‐AOC) (FRAP method), thiobarbituric acid reactive substance, 2,2′‐azino‐bis(3‐ethylbenzothiazoline‐6‐sulfonic acid) radical scavenging, and 2,2‐diphenyl‐1‐(2,4,6‐trinitrophenyl) hydrazyl (DPPH) radical scavenging. Our results indicated that tea polyphenols have a great antioxidant capacity and that a high frying temperature causes fat oxidation. Our study confirmed that DPPH assay is more suited to lipophilic compounds or compounds with high lipid content. In a frying temperature of 180°C, the DPPH‐free radical scavenging ability of pork was not decreased. Further experiments remain necessary to explore specific temperatures with the same results. This study provides new process parameters and new references for processing techniques of healthy and high‐quality pork products.

## Graphical abstract

Tea polyphenols added to pork have significant antioxidant capacity for frying at different temperatures and times. The following findings were revealed. Our study confirmed that DPPH assay was more suited to lipophilic compounds or compounds with high lipid content, but the frying temperature should not be too high; in this study, the DPPH‐free radical scavenging ability of pork did not decrease at 180°C. Further experiments are warranted to explore specific temperatures with the same results.

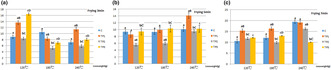

## 1 INTRODUCTION

Meat is the muscle tissue of slaughtered animals, which is composed of water, proteins, lipids, minerals, and a small proportion of carbohydrates. Meat and meat products are susceptible to quality deterioration because of their rich nutritional composition (Devatkal et al., 2014). Because of the presence of fat, meat and meat products are unstable, with easy deterioration and undergoing changes during storage and processing, primarily oxidation. These changes, apart from microbial growth, are the main cause of the limited shelf‐life of meat products. Compounds that form as a result of auto‐oxidation may have an adverse effect on the quality and flavor of the final product (Ahn et al., 2001; Ahn & Nam, 2008; Frankel, 2001).

Change in the antioxidant capacity is one of the major causes of deterioration in food products, leading to unfavorable changes in flavor, texture, and color. Oxidation damages the nutritional quality of foods through the degradation of vitamins and essential fatty acids such as linoleic and linolenic acids (Kirk, 1984). Therefore, both foods and the human body must be protected from excessive oxidation. One way of achieving this is by the addition or preservation of existing antioxidants. Tea polyphenols have a phenolic hydroxyl structure, which reduces the binding ability of hydrogen ions. Active hydrogen ions directly scavenge free radicals and other active oxygen species (Zuo et al., 2018). Their *ortho*‐diphenol hydroxyl group can also become a reaction site for metal chelates, significantly reducing the oxidative stress of high concentrations of carbonyl groups (Yadvdk, 2021). They maintain and repair the antioxidant system of cells, thereby effectively preventing tumors, antiaging, and cardiovascular and cerebrovascular diseases. Various in vitro measurement methods were used to detect the changes in antioxidants of pork added with different concentrations of tea polyphenols under different cooking methods. In this study, the antioxidant capacity of different concentrations of tea polyphenols at different times and temperatures of pork was examined by using several assays to test for different antioxidant mechanisms (iron chelation, 2,2′‐azino‐bis(3‐ethylbenzothiazoline‐6‐sulfonic acid) [ABTS] radical scavenging, 2,2‐diphenyl‐1‐(2,4,6‐trinitrophenyl)hydrazyl [DPPH] radical scavenging, and lipid oxidation in emulsion) to assess (1) the capacity of tea polyphenols as an antioxidant; (2) differences between different times and temperatures; and (3) the underlying mechanism of any potential antioxidant capacity.

There are different relevant process technologies and process parameters, including process time and process temperature. The antioxidants in meat products are influenced significantly by these elements. This study explored if the antioxidant capacity of meat and the nutritional quality of meat products are affected by temperatures, times, and other factors. The change rules of antioxidants in meat products were examined in process methods and process parameters. The quality of meat and meat products improved, which provided a theoretical basis about antioxidation for meat.

## 2 MATERIALS AND METHODS

### 2.1 Chemicals

DPPH and ABTS were purchased from Sigma‐Aldrich (Steinheim, Germany). Vitamin C, ethanol, trichloroacetic acid, NaCl, and ferricyanide hydrochloride were purchased from National Medicines Corporation, Ltd. (China).

### 2.2 Cooking of chilled pork

Chilled pork was sent to the laboratory in a refrigerated box. The treated pork measured 10 × 1.5 × 1 cm. Then, experimental requirements were performed based on different times, temperatures, and other factors.

The C group (control group) was natural chilled pork. The TP1 group was treated with 6 g/L of tea polyphenols 1 L at 4°C overnight before cooking. The TP2 group was treated with 9 g/L of tea polyphenols 1 L at 4°C overnight before cooking. The TP3 group was treated with 12 g/L of tea polyphenols 1 L at 4°C overnight before cooking.

Each experimental group had different time intervals (i.e., 3, 5, and 7 min) and temperatures (i.e., 120, 180, and 240°C) (as shown in Table 1).

**TABLE 1:** Experimental plan table.

Tea polyphenol concentration (g/L)	Temp (°C)	C	TP1	TP2	TP3
Time (min)					
3	120	0	6	9	12
	180	0	6	9	12
	240	0	6	9	12
5	120	0	6	9	12
	180	0	6	9	12
	240	0	6	9	12
7	120	0	6	9	12
	180	0	6	9	12
	240	0	6	9	12

The treated pork was weighed, and then four times of deionized water was added. Pork strips were high‐speed sheared in an ice water bath. The meat cells were broken to release the antioxidants. The centrifuge was adjusted to 4°C, 15,294 ***g***; centrifugation was performed for 5 min. The supernatant was obtained and kept frozen until further analysis.

### 2.3 Chemical analyses

The total antioxidant capacity (T‐AOC) of pig meat was assayed using the T‐AOC kit (FRAP method). Fat oxidation is an important factor affecting pig meat quality and freshness. A thiobarbituric acid reactive substance (TBA) assay was extensively used to evaluate the degree of fat oxidation in meat products. As an important parameter, it reflects the degree of fat oxidation. A modified version of the TBARS procedure by Siu and Draper (1978) was used to measure lipid oxidation. Total protein was determined colorimetrically (Bradford et al., 1976). The weight loss in cooking was determined by weighing.

### 2.4 Ferric reducing antioxidant power (FRAP) assay

The meat samples were weighed and mixed with four times the volume of physiological saline or PBS. This mixture was mechanically homogenized under ice water bath conditions to fully crush cells and release antioxidants. Subsequently, the slurries were centrifuged at 4°C at 12,000 rpm for 5 min, and the supernatant was collected and assayed for T‐AOC.

The FRAP reagent contained 2.5 mL of 10 mM 1,3,5‐Triazine (TPTZ) solution in 40 mM HCl, along with 2.5 mL of 20 mM FeCl_3_ and 25 mL of 0.3 M acetate buffer at a pH of 3.6, and it was freshly prepared and warmed at 37°C. After adding the treated samples and incubating them at 37°C for 5 min, the absorbance of the reaction mixture was measured at 593 nm. The final results were expressed as the concentration of antioxidants with a ferric reducing ability equivalent to that of 1 mM FeSO_4_ based on the standard curve for FeSO_4_.7H_2_O (at a concentration range between 100 and 1000 mM).

### 2.5 T‐AOC assay standard curve

#### 2.5.1 MDA assay (TBA method)

**FIGURE 1:**
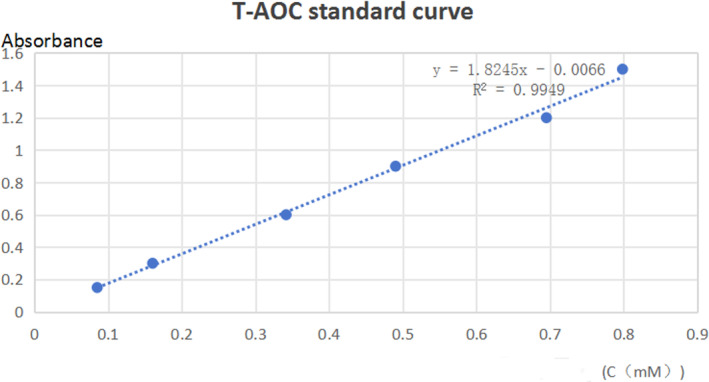
T‐AOC assay standard curve.

Malondialdehyde (MDA), the final product of the lipid peroxidation reaction in organisms, can result in the cross‐linking polymerization of organic macromolecules such as proteins and nucleic acids. MDA is an important target that reflects the body's antioxidant potential, lipid peroxidation rate, and intensity, as well as indirectly reflecting the degree of tissue peroxidation damage (Figure 1).

In the experimental procedure, 10 mg of tissue was treated with 500 μL of moderate strength RIPA cracking solution (supplemented with 100 × BHT) in an ice bath. After homogenization, the homogenate was centrifuged at 4°C for 10 min at 12,000 rpm. The resulting supernatant was used for MDA determination.

To prepare the standard solution, 50 μL of 1 mM standard was added to 950‐μL water, resulting in an MDA concentration of 50 μM. Subsequently, five additional 1.5‐mL centrifuge tubes were prepared. To each tube, 500‐μL water was added, followed by the addition of 500 μL of 50‐μM MDA standard concentration, which was then diluted to concentrations of 25, 12.5, 6.25, 3.12, and 1.56 μM, respectively (as shown in Table 2).

**TABLE 2:** MDA assay implementation table.

	Blank tube (μL)	Standard tube (μL)	Sample tube (μL)	Control tube (μL)
Water	100			200
Standard		100		
Sample			100	100
SDS	100	100	100	100
TBA	200	200	200	

These tubes were then boiled in a water bath for 30 min and cooled to room temperature in an ice bath. Afterward, the tubes were centrifuged at 10,000 × *g* for 10 min, 200 μL of supernatant was collected, and the absorbance was measured at 535 nm.

### 2.6 MDA assay standard curve

#### 2.6.1 DPPH‐free radical scavenging ability

**FIGURE 2:**
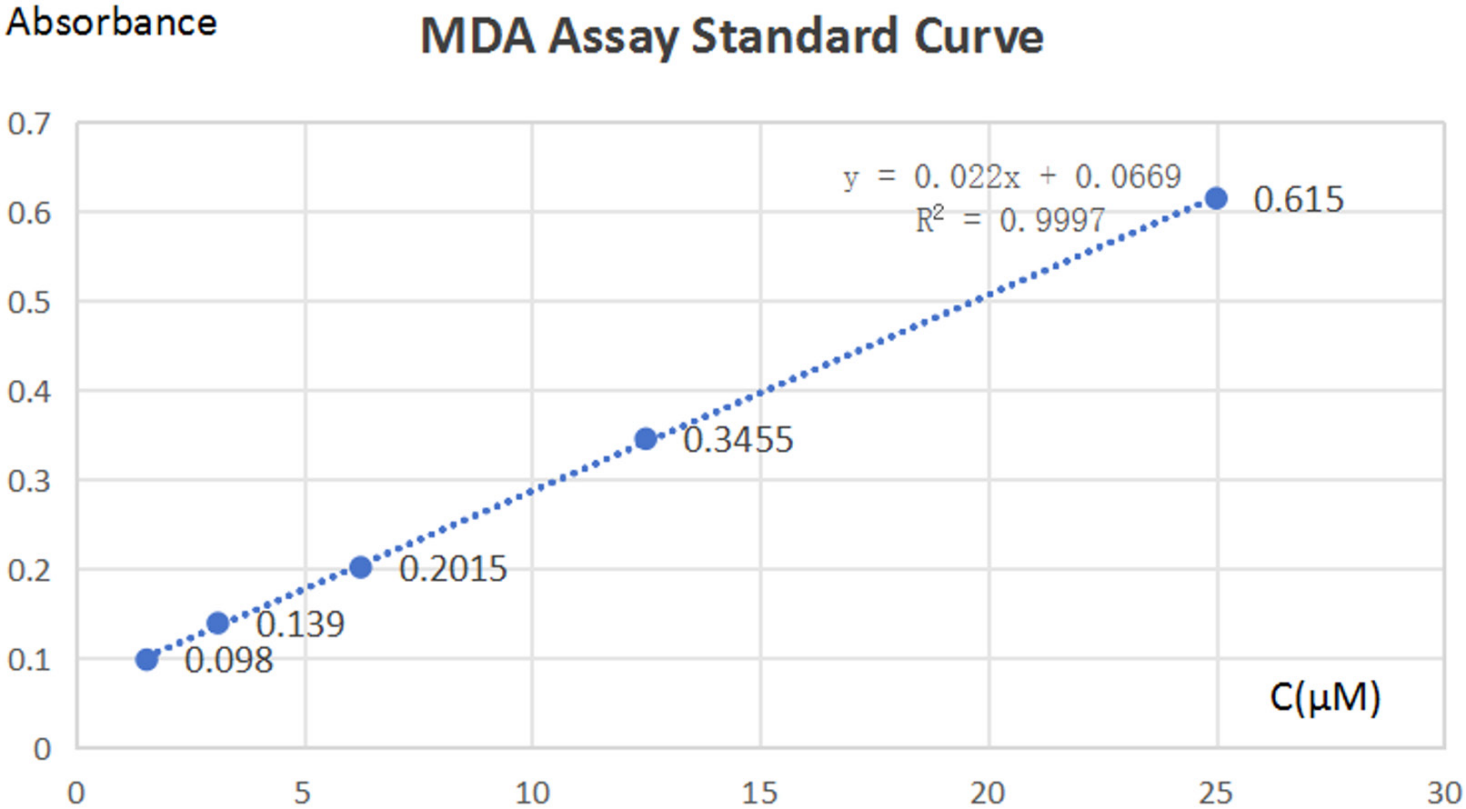
MDA assay standard curve.

The Brand–William method was used in this experiment (Bondet et al., 1997) (Figure 2).

Prepare 257.7 mg/L of DPPH stock solution (95% ethanol as solvent) with different concentrations (62.5, 125, 250, 500, and 1000 μg/mL) of sample solution, vitamin C solution, and 2,6‐di‐*tert*‐butyl‐*p*‐cresol solution for standby.

Prepare 0.4 mL of sample solution with different concentrations. Add 4.0 mL of 257.7 mg/L of DPPH and mix well. Place the solution in a 37°C water bath for 60 min. Measure the absorbance at 517 nm, and record it as sample *A*_s_.

Prepare 0.4 mL of 95% ethanol. Add 4.0 mL of 257.7 mg/L of DPPH and mix well. Place the solution in a 37°C water bath for 60 min. Measure the absorbance at 517 nm, and record it as blank *A*_b_.

The absorbance value of vitamin C and 2,6‐di‐*tert*‐butyl‐*p*‐cresol was measured according to the aforementioned method as a control.

(1) $$\text{DPPH scavenging ability}\;\left(\%\right)=\frac{A_{b}-A_{s}}{A_{b}}\times 100\%$$

### 2.7 ABTS ^+^ free radical scavenging ability

The radical scavenging capacity of the hydrolysates was assayed with ABTS^+^ according to the protocol of Jensen et al. (2011). The ABTS^+^ radical solution (19.4 mmol/L of ABTS^+^ and 6.7 mmol/L of potassium persulfate) was diluted with 10 mmol/L of phosphate buffer, pH 7.4, until A_405nm_ reached 0.7. All samples were filtered using a 0.45‐μm filter and diluted with distilled water to 50 μg/mL. Samples of 50 μL were subsequently mixed with 200 μL of ABTS^+^ radical solution, and the absorbance of the resulting mixtures was measured after 1 h at 405 nm. The scavenging capacity was calculated by the following equation:

(2) $$\text{Radical scavenging}\;\left(\%\right)=100-\left(100\times \frac{A_{\text{sample}}-A_{\text{blank}}}{A_{\text{control}}}\right)$$

where *A*_sample_ is the absorbance of the ABTS^+^ mixed with the sample, *A*_control_ is the absorbance of the ABTS mixed with water, and *A*_blank_ is the absorbance of the sample mixed with water. All measurements were performed in triplicate and reported as the average value. Trolox (32 μmol/L) was used as a reference.

### 2.8 Statistical analysis

In the present study, hypothesis testing methods included ANOVA and least significant difference testing. *p* < .05 was considered to be statistically significant. All analyses were carried out using SPSS 21 software (SPSS Inc., Chicago, IL, USA).

## 3 RESULTS AND DISCUSSION

In this study, chilled pork from each experimental group was fried at different times and temperatures (Table 1).

### 3.1 Antioxidant capacity of tea polyphenols in pork for frying

**FIGURE 3:**
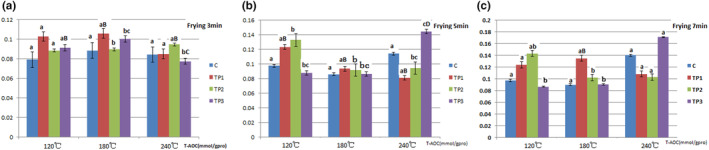
Effect of tea polyphenols on T‐AOC due to frying at different temperatures and times for pork samples. The data points without same superscripts (A, B) are significantly different (one‐way ANOVA). Letters represent differences in different groups, such as capital letters at the level of *p* < .01 by least significant difference (LSD) test; Lower case letter at the level of *p* < .05 by least significant difference (LSD) test. C group: Control group, the natural chilled pork; TP_1_ group: in 6 g/L of tea polyphenols 1 L at 4°C overnight before cooking; TP_2_ group: in 9 g/L of tea polyphenols 1 L at 4°C overnight before cooking; TP_3_ group: in 12 g/L of tea polyphenols 1 L at 4°C overnight before cooking.

**FIGURE 4:**
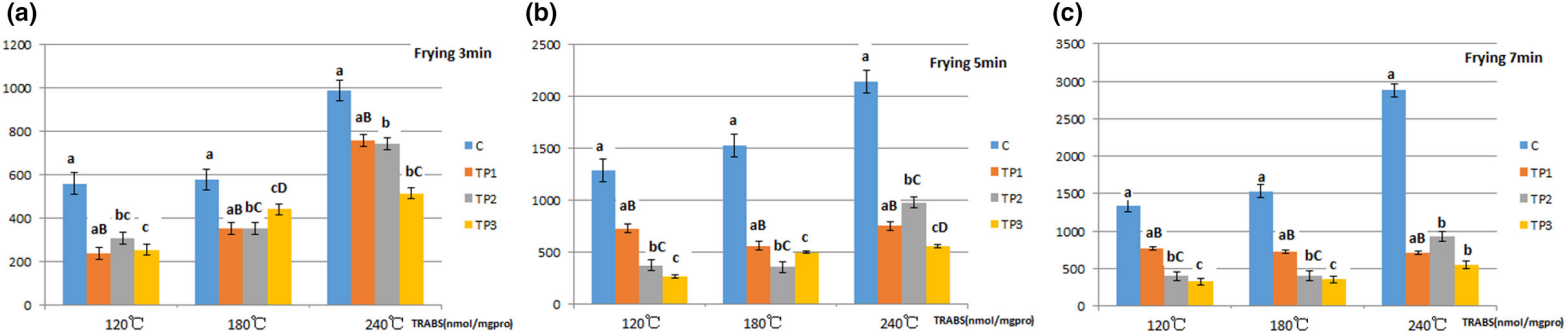
Effect of Tea Polyphenols on TRABS due to frying at Different temperatures and times for pork samples. The data points without same superscripts (A, B) are significantly different (one‐way ANOVA). Letters represent differences in different groups, such as capital letters at the level of *p* < .01 by least significant difference (LSD) test; Lower case letter at the level of *p* < .05 by least significant difference (LSD) test. C group: Control group, the natural chilled pork; TP_1_ group: in 6 g/L of tea polyphenols 1 L at 4°C overnight before cooking; TP_2_ group: in 9 g/L of tea polyphenols 1 L at 4°C overnight before cooking; TP_3_ group: in 12 g/L of tea polyphenols 1 L at 4°C overnight before cooking.

The antioxidant capacities of tea polyphenols in pork for frying are presented in Figures 3 and 4.

### 3.2 Total antioxidant capacity

The FRAP method is based on an oxidation–reduction reaction. At an acidic pH value, Fe^3+^ forms a complex with TPTZ (Fe^3+^–TPTZ). Under the action of reducing substances, the complex is reduced to Fe^2+^ and appears blue. The maximum absorption was reached at 593 nm, and the change in absorbance was proportional to the content of reducing substances. It can be used as a method to determine the total antioxidant capacity of substances.

**FIGURE 5:**
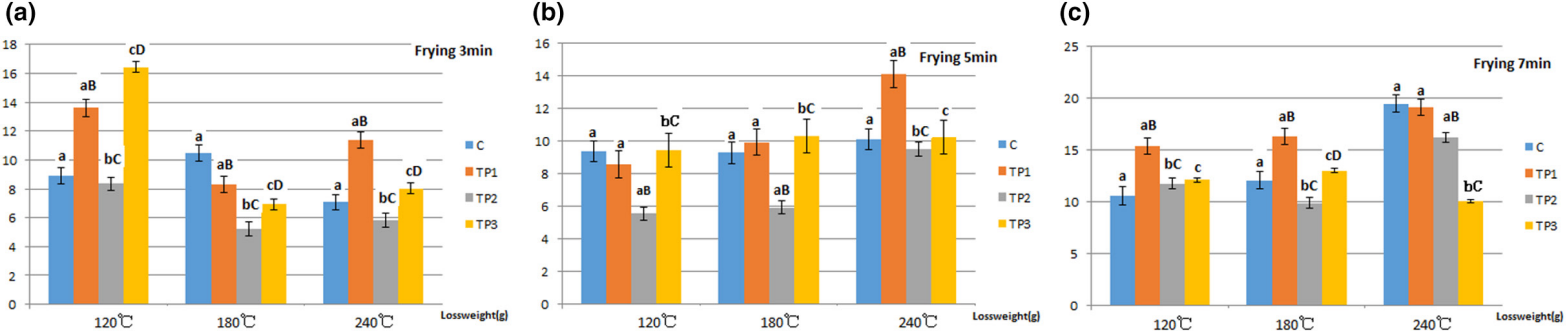
Effect of tea polyphenols on weight loss at different temperatures and times for pork samples. The data points without same superscripts (A, B) are significantly different (one‐way ANOVA). Letters represent differences in different groups, such as capital letters at the level of *p* < .01 by least significant difference (LSD) test; Lower case letter at the level of *p* < .05 by least significant difference (LSD) test. C group: Control group, the natural chilled pork; TP_1_ group: in 6 g/L of tea polyphenols 1 L at 4°C overnight before cooking; TP_2_ group: in 9 g/L of tea polyphenols 1 L at 4°C overnight before cooking; TP_3_ group: in 12 g/L of tea polyphenols 1 L at 4°C overnight before cooking.

In this study, compared with the C group, tea polyphenols added in pork resulted in increased T‐AOC at 120°C. The TP3 group exhibited a significant T‐AOC increase at 120°C. At 180°C, an increase in tea polyphenols causes an increase in T‐AOC. At 240°C, an increase in tea polyphenols causes a decrease in T‐AOC, which may be due to a significant increase in weight loss. For example, in this study, compared with the C group, the TP3 group had a weight loss of 93.28% at 240°C of 7‐min frying. Our study indicated that at 180°C, 12 g/L of tea polyphenols can counteract the reduction of T‐AOC. Further, at 240°C, 12 g/L of tea polyphenols cannot counteract the reduction of T‐AOC (Figure 5).

### 3.3 Lipid oxidation: thiobarbituric acid reactive substance method

Tea polyphenols can inhibit the growth of microorganisms in meat products, delay fat oxidation, and extend shelf life. The TBA value reflects the degree of antioxidant activity: the lower the TBA value, the better the oxidative stability of muscles, and the lesser the loss of nutrients in muscles. Studies (Cimmino et al., 2018; Liu et al., 2018) had suggested that in lamb, plant polyphenols reduce the TBA value, inhibit fat oxidation, and improve the quality of meat. Plant polyphenols with antioxidant properties can reduce the loss of nutrients in meat products and ensure the freshness and quality of meat products.

In this study, the TBA value in the frying of pork at various temperatures and times was significantly decreased because of the added tea polyphenols in pork. The TBA value more significantly decreases at higher temperatures. These indicated that tea polyphenols have significant antioxidant activity.

### 3.4 DPPH and ABTS ^+^ free radical scavenging ability

Polyphenol compounds are good antioxidants, and their antioxidant effect is closely related to the easy oxidation of *ortho*‐phenolic hydroxyl groups in the chemical structure. Phenolic hydroxyl groups can provide active hydrogen to inactivate free radicals, which are oxidized to form free radicals containing *ortho*‐diphenol structures and have high stability (Yang et al., 2016). This study evaluated the antioxidant activity of tea polyphenols through DPPH radical and ABTS^+^ radical scavenging. The results indicated that the scavenging ability of tea polyphenols to DPPH radical and ABTS^+^ radical was concentration dependent within a certain range of mass concentration.

**FIGURE 6:**
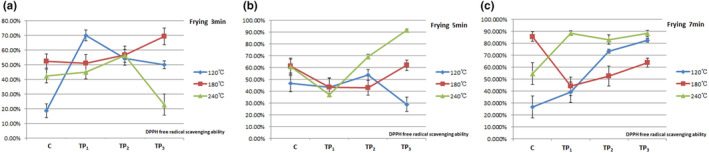
Effect of tea polyphenols on DPPH free radical scavenging ability at different temperatures and times for pork samples. The data points without same superscripts (A, B) are significantly different (one‐way ANOVA). Letters represent differences in different groups, such as capital letters at the level of *p* < .01 by least significant difference (LSD) test; Lower case letter at the level of *p* < .05 by least significant difference (LSD) test. C group: Control group, the natural chilled pork; TP_1_ group: in 6 g/L of tea polyphenols 1 L at 4°C overnight before cooking; TP_2_ group: in 9 g/L of tea polyphenols 1 L at 4°C overnight before cooking; TP_3_ group: in 12 g/L of tea polyphenols 1 L at 4°C overnight before cooking.

The lipid peroxide free radicals generated during high‐temperature cooking of fats were the main risk factors for cardiovascular and cerebrovascular diseases and tumors. Therefore, the oil temperature should be less than 150°C during frying. Food should not be cooked at high temperatures or for extended periods. The ABTS^+^ and DPPH radical scavenging assays were used in our study. Because both assays are based on electron transfer mechanisms involving the reduction in colored pro‐oxidants, they would be expected to yield similar results. DPPH‐free radical scavenging ability was significantly decreased in TP3 at 240°C of 3‐min frying; greatly decreased in TP3 at 120°C of 5‐min frying; 22.81% at 240°C of 3‐min frying; and 28.97% at 120°C of 5‐min frying (Figure 6). We believe that high frying temperature causes fat decomposition. DPPH assay was performed in an organic solvent system; it is more suited to lipophilic compounds or compounds with high lipid content, which was confirmed in our study. The frying temperature should not be too high; in this study, the DPPH‐free radical scavenging ability of pork did not decrease at 180°C. Further experiments remain necessary to explore specific temperatures with the same results (Figure 6).

**FIGURE 7:**
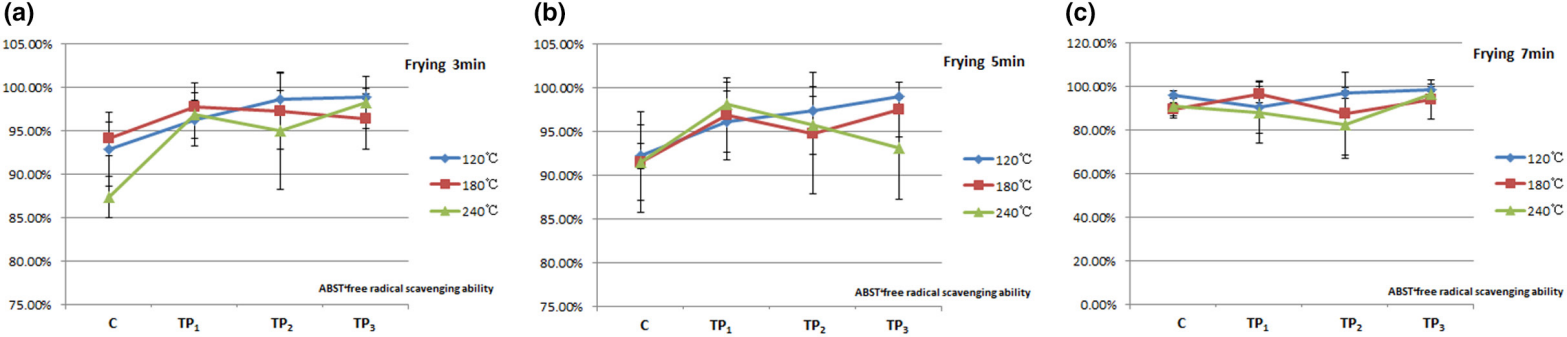
Effect of tea polyphenols on ABTS^+^ free radical scavenging ability at different temperatures and times for pork samples. The data points without same superscripts (A, B) are significantly different (one‐way ANOVA). Letters represent differences in different groups, such as capital letters at the level of *p* < .01 by least significant difference (LSD) test; Lower case letter at the level of *p* < .05 by least significant difference (LSD) test. C group: Control group, the natural chilled pork; TP_1_ group: in 6 g/L of tea polyphenols 1 L at 4°C overnight before cooking; TP_2_ group: in 9 g/L of tea polyphenols 1 L at 4°C overnight before cooking; TP_3_ group: in 12 g/L of tea polyphenols 1 L at 4°C overnight before cooking.

The ABTS^+^ assay is compatible with both aqueous and organic solvent systems (Arnao et al., 2001). Other studies have compared the two assays, and the antioxidant capacity detected by the ABTS^+^ assay has been reported to be significantly higher for a variety of foods when compared with that of the DPPH assay, partly because the highly pigmented and hydrophilic antioxidants are better reflected by the ABTS^+^ assay than the DPPH assay (Damgaard et al., 2014; Floegel et al., 2011; Kim et al., 2002), suggesting that the ABTS^+^ assay may be better than the DPPH assay for detecting the antioxidant capacity in a variety of foods (Figures 6 and 7). Their results were consistent with the results of this study.

## 4 CONCLUSION

Our study indicated that tea polyphenols added to pork have significant antioxidant capacity for frying at different temperatures and times. The following findings were revealed. (1) At 240°C, an increase in tea polyphenols caused a decrease in T‐AOC, which may be due to a significant increase in weight loss. Alexandre et al. (2022) found that green tea extracts had no significant effect on lipid oxidation delay. Interestingly, treatments with added green tea extracts exhibited the highest TBARS values (*p* ≤ .05) in the tilapia meat, measuring 24.22 units higher than those of the control after 180 days of storage. It is possible that the concentration of green tea extract might not have been sufficient to neutralize the oxidizing factors present in tilapia meat, potentially causing the extracts to act as pro‐oxidants. Our study, which involved treating meat at different temperatures, yielded different results, aligning with the findings reported by Alexandre et al. (2022). At 180°C, 12 g/L of tea polyphenols could counteract the reduction of T‐AOC. However, at 240°C, the same concentration of 12 g/L of tea polyphenols could not counteract the reduction of T‐AOC. DPPH‐free radical scavenging ability in TP3 was significantly decreased at 240°C of 3‐min frying; and greatly decreased at 120°C of 5‐min frying. Accordingly, high frying temperature caused fat decomposition. Our study confirmed that DPPH assay was more suited to lipophilic compounds or compounds with high lipid content, but the frying temperature should not be too high; in this study, the DPPH‐free radical scavenging ability of pork did not decrease at 180°C. Further experiments are warranted to explore specific temperatures with the same results.

## AUTHOR CONTRIBUTIONS

**WeiJiang Fan:** Data curation (equal); writing – original draft (equal); software (equal); funding acquisition (equal). **GuoHui Du:** Data curation (equal). **XueNa Zhang:** Data curation (equal). **ShuQing Wang:** Resources (equal). **FeiHong Long:** Software (equal). **Chao Li:** Software (equal). **NingPeng Jiang:** Software (equal). **Yinglong Zhang:** Software (equal). **Qiang Sun:** Software (equal).

## CONFLICT OF INTEREST STATEMENT

The authors declare that they have no known competing financial interests or personal relationships that could have appeared to influence the work reported in this paper.

## Data Availability

The authors confirm that the data supporting the findings of this study are available within the article.
